# Detecting Cancer Gene Networks Characterized by Recurrent Genomic Alterations in a Population

**DOI:** 10.1371/journal.pone.0014437

**Published:** 2011-01-04

**Authors:** Sol Efroni, Rotem Ben-Hamo, Michael Edmonson, Sharon Greenblum, Carl F. Schaefer, Kenneth H. Buetow

**Affiliations:** 1 The Mina & Everard Faculty of Life Science, Bar Ilan University, Ramat Gan, Israel; 2 Laboratory of Population Genetics, National Institutes of Health, Bethesda, Maryland, United States of America; 3 National Cancer Institute Center for Biomedical Informatics and Information Technology, National Institutes of Health, Bethesda, Maryland, United States of America; Massachusetts General Hospital, United States of America

## Abstract

High resolution, system-wide characterizations have demonstrated the capacity to identify genomic regions that undergo genomic aberrations. Such research efforts often aim at associating these regions with disease etiology and outcome. Identifying the corresponding biologic processes that are responsible for disease and its outcome remains challenging. Using novel analytic methods that utilize the structure of biologic networks, we are able to identify the specific networks that are highly significantly, nonrandomly altered by regions of copy number amplification observed in a systems-wide analysis. We demonstrate this method in breast cancer, where the state of a subset of the pathways identified through these regions is shown to be highly associated with disease survival and recurrence.

## Introduction

Biologic phenotypes emerge as a consequence of genes interacting through complex networks. Oncogenesis has been shown to be dependent on biologic networks that control processes such as apoptosis, senescence, proliferation, and angiogenesis [1], [2]. However, it is clear that current knowledge of which processes influence diverse cancer phenotypes is incomplete. This is especially true when it comes to understanding processes associated with disease outcome.

A complex collection of genomic alterations occur during tumor cell evolution, including mutations, translocations, and copy number alterations. For example, genome-wide analysis of breast tumors by numerous techniques have reproducibly demonstrated recurrent patterns of copy number alteration (CNA) [3], [4], [5], [6], [7], [8], [9], [10], [11]. The expression of genes within these altered segments has been demonstrated to be correlated with the copy number state of the region [3], [9], [12], [13], [14], [15], [16], [17], [18], [19]. However, it is unclear whether these recurrent patterns represent the most important set of CNAs or represent only a subset of key regions.

Patterns of copy number alteration have proven valuable in classification of cancer subtypes and can serve as predictors of patient outcome [19]. These alterations target genes that influence networks that provide the tumors with a selective advantage over cells of normal composition. Given their association with outcome, it is likely they also influence processes that drive clinical phenotypes and response to interventions.

Identifying the processes targeted by the regions identified through system-wide analysis is complex. For example, copy number-altered regions contain large numbers of genes. There is also a tremendous degree of between-individual heterogeneity in the inventory of regions found to be altered.

Work by others to identify processes underpinning complex traits has combined inherited variants and network analysis to map multifactorial, heterogeneous disease phenotypes [20]. In this work, the authors extend traditional gene mapping approaches by including putative gene interactions to address heterogeneity. Others have examined multidimensional data sets that include different genome-scale measurements simultaneously in the context of pathways [21], [22], [23].. They apply statistical method to measure pathway enrichment and use gene-expression data to assess variation of pathway activity. Through such analyses they hypothesize new cell functions.

In the work presented here, we compliment and extend these approaches to systematically analyze somatic CNAs to identify biologic networks underpinning cancer phenotypes. We demonstrate the method using the breast cancer data set of Chin et al [24]. We identify altered pathways differentially targeted by copy number aberrations.

Similar to previous approaches, we addresse the heterogeneity of patterns by recognizing that differing patterns of CNA may represent alternative routes that cancer cells may take to alter the same core set of common biologic processes. The apparent heterogeneity in map location associated with CNAs may simply reflect the fact that the genes comprising a given network are distributed throughout the genome. We therefore test whether individual canonical pathways are non-randomly targeted across copy number change regions. In contrast to previous approaches, we leverage existing network structure as opposed to de novo creating networks. The network interaction structure for these canonical networks is then leveraged for mapping phenotypes. We utilize previously described methods [25] to determine whether altered state of non-randomly altered processes can predict patient outcome.

## Results

Chin et al. have previously reported genome-wide copy number and gene expression analysis of 145 primary breast cancer tumors [19]. These alterations were determined using genome BAC array CGH [26], [27], [28], [29] comprised of 2464 BACs selected at approximately mega base intervals along the genome as described previously [26], [28]. Utilizing this data set and the process described in **Materials and Methods**, the gene content of each segment described in Chin et al. was identified.

Canonical biologic network structure information and gene content was obtained from public sources [30], [31], [32].A total of 565 canonical pathways were examined. These pathways represent collections of interactions that are subsets of larger biologic networks curated to capture specific functions. Therefore, their gene content is not unique. The gene content of these pathways ranges dramatically. For example, as the pathway “degradation of the RAR and RXR by the proteasome [33]) contains only 2 genes while IL12 Signaling Pathway” [34], [35], [36])contains 80.

To account for heterogeneity of gene involvement when analysis is performed using a network model we define a new statistical metric (described in equations (2.5) and (2.6) in **Materials and Methods**). Significance for each pathway across samples was assessed using the Fisher's Omnibus [49] and adjusted for multiple comparisons using the Bonferoni method.

Applying the methods to the data provided by Chin et al., we identify pathways in which the genes altered by CNAs are highly significantly over-represented when compared to random expectations (Table S1).

**Figure 1:**
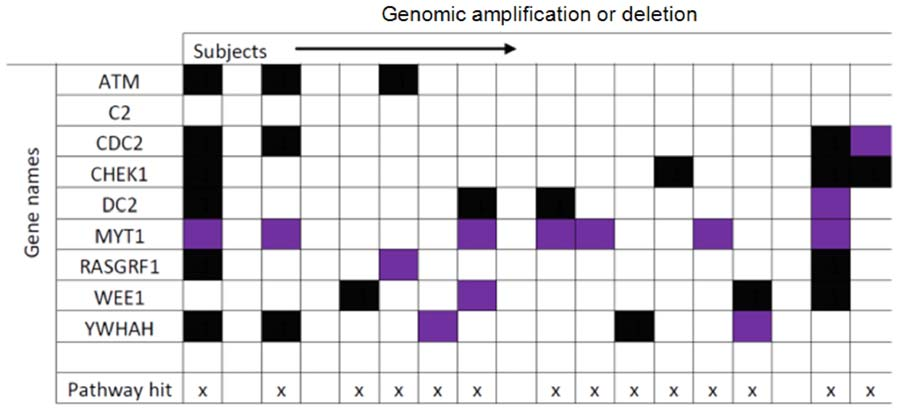
Copy Number alterations in 18 subjects in the “CDC25 and CHK1” pathway. Purple rectangles signify gene amplification and black squares signify deletion. Each column represents a randomly chosen subject with a total of 18 subjects. Each row represents a different gene of the pathway genes. Different subjects target the “CDC25 and CHK1” pathway through alternating genomic strategies. The pathway as a unit, however, is targeted throughout the population.

To illustrate the diverse over-representation patterns for a given network we present the CNA events associated with the pathway “CDC25 and CHK1” [37] (Figure 1). In the figure, gene amplification is denoted through a purple square and gene deletion through black squares.

As Figure 1 demonstrates, no single gene within the pathway appears to be the differential target of CNA across the 18 breast cancer samples shown… or when examined across the remaining 127 individuals in the study.

On the other hand, we can see that the pathway, as a unit, is targeted in almost every subject in the panel (the entire panel of subjects for this pathway is included in Table S2). Note, the metric (see Materials and Methods) **compensates for pathway size.** As such, to obtain a significant p-value, larger pathways need to accumulate a larger number of gene amplifications or deletions.

**Figure 2:**
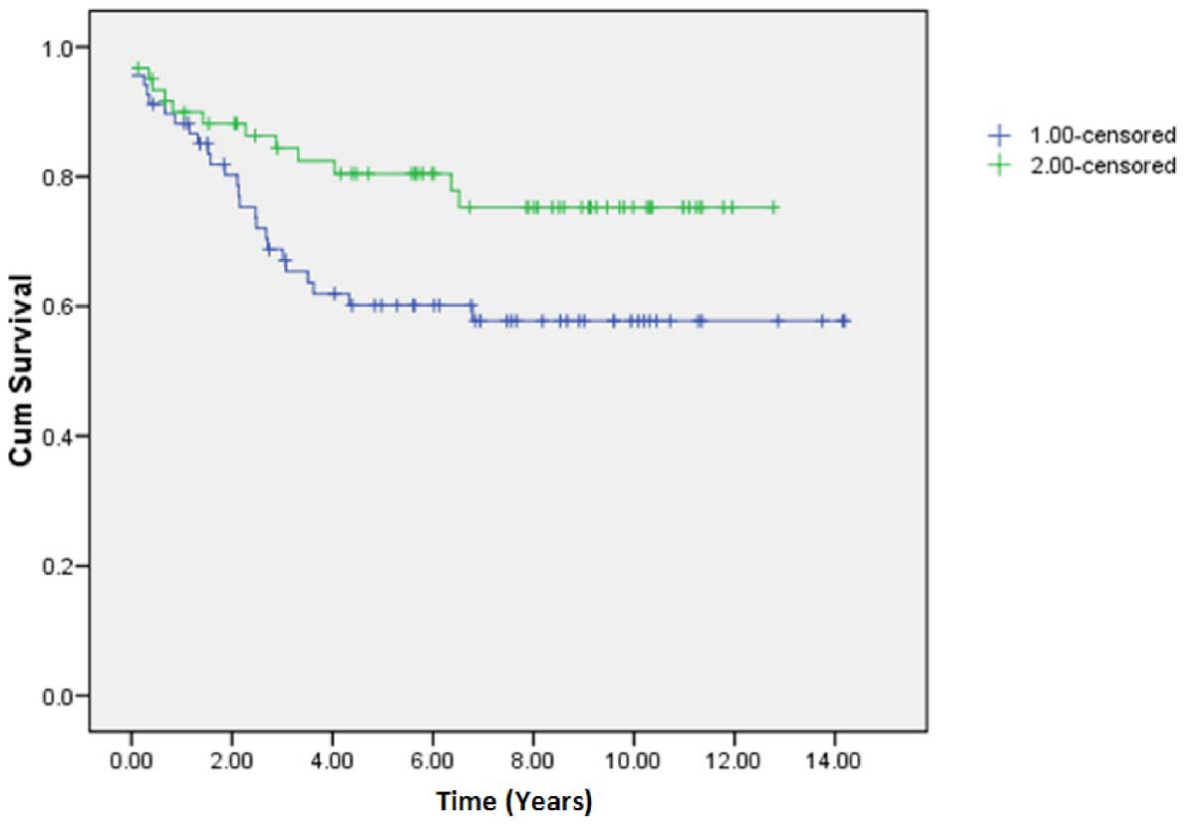
Kaplan-Meier survival curve of the “CDC25 and CHK1” pathway (P-value = 0.04). This pathway, which has been highlighted through its highly significant p-value as targeted by genomic alterations, is highly significant in its ability to stratify patients' prognosis. The figure demonstrates how significant genomic alterations indicate a pathway's significance as a stratification tool.

We next assessed whether the networks identified by over-representation of CNA are associated with disease outcome. Using pathway activity and pathway consistency scores [26], we clustered the individuals according to their pathway metrics and performed survival analysis. When we stratify the patients to two groups, we can draw the survival curves and check to see if they separate the population in a significant manner (Figure 2).

Iterating over the collection of hundreds of pathways, we find 29 pathways that meet significance criteria of p<0.05 (Table S3). However when adjusting for multiple testing using the Bonferroni method only two pathways significantly targeted by genomic alterations are also highly associated with survival;”“Hypoxic and oxygen homeostasis regulation of HIF-1-alpha” [38], [39], [40], and Glycosaminoglycan degradation [refs].

An alternative approach to adjusting for multiple comparisons for assessing significance is to validate findings those pathways that show marginal significance across data sets. Two public data sets with expression data and disease outcome were selected from the Gene Expression Omnibus database (http://www.ncbi.nlm.nih.gov/geo) [41] The first data set (GSE2990) [42] contained 189 individuals. The second (GSE3494) [43] contained 251 individuals. Gene expression in both datasets utilized the Affymetrix platform for determining gene expression state. Of the original 29 pathways observed to be significantly associated with survival in Chin et al. [19], 8 were observed to be significant in GSE2990 and 8 were observed to be significant in GSE3494. A total of 4 pathways were observed to be significant in all three data sets. Concordance among the datasets is more than would be expected by chance alone.

## Discussion

The above results suggest that genes in CNA non-randomly target processes important for oncogenic state. In the work presented here, we provide a means for objectively identifying the biologic processes that may be the target of these alterations. Moreover, the pathways over-represented in these segments show differences in activity and consistency that is related to cancer outcome.

The total number of pathways identified as non-randomly targeted is striking. One possible explanation is the lack of independence of the gene content associated with each pathway. Hierarchical clustering of the pathways utilizing the p-value associated with the non-random targeting (Table S4) confirms that pathways with related names commonly cluster with high correlation (r>0.5, data not shown). Inspection of the pathway p-values across individuals shows tremendous variability (Table S4). This suggests diverse underlying molecular mechanisms driving oncogenesis. Unfortunately, no obvious pattern of clustering of individuals emerges from analysis of pathway-specific variability.

CNA have been previously demonstrated to show association with patient outcome [44], [45], [46], [47]). In the Chin et al. [19] individual copy number altered segments showed association with survival and disease recurrence, but performed unevenly. When taken as a set, they found that alteration of any of what they identified as “recurrent amplicons” was associated with reduced survival duration (p<0.04) and distant recurrence (p<0.01).

The results obtained from pathway-based analysis of the same data set produce a striking improvement and suggest that pathways may represent a better way to evaluate recurrent alterations. Two pathways show a highly significant association within Chin et al. alone and 4 pathways show significance across multiple data expression datasets. Because of the high dimensionality of systems-wide data, there is always a danger of over fitting. As such, results from an individual study should be viewed skeptically. However, the significant concordance across multiple provides independent validation.

The increased reproducibility and magnitude of the effect associated with pathway state compared with that observed in the direct examination of “recurrent” regions may be attributable to several factors. At a mechanical level, examination of data at the pathway level permits the information from different regions to be integrated across the network. The fact that any given recurrent region is amplified is no longer the critical predictor. What emerges instead is the importance of sets of altered regions whose individual members hit different parts of a targeted pathway. Pathways pre-aggregate the effects of multiple genes. As such, it is possible to detect multigene interactions that influence cancer phenotypes but which, if not aggregated in a pathway, might fail to meet the test of statistical significance in a small dataset.

CNA is only one factor that could be driving pathway involvement in phenotypes. Many other genomic mechanisms (e.g. individual gene mutations, epigenetic activation/silencing) can influence the state of the pathway. As such, the pathways identified here represent a subset of those likely involved.

Conceptually, it is likely that because the pathway is the underlying unit of the phenotype, focusing on pathways increases signal and reduces noise. Genomic alterations that accumulate during oncogenesis and disease progression occur at random. The observed coherence likely arises because certain processes must be altered to arrive at the given phenotype. Apparent genomic heterogeneity, “noise”, arises because there are multiple ways a pathway can be changed. All of these ways are “signal” from the perspective of a pathway.

It is possible to speculate that analysis similar to those performed for copy number alteration to pathway (above) may prove useful for other genome analyses such as genome-wide mutational screens or association studies. For example, the complex mutational patterns seen in the 1672 genes characterized in human and breast cancer [48] are all observed to mutate genes in one or more of 6 canonical pathways state identified from gene expression data which universally differentiates tumor from normal [25]. Similarly, complex, low odd-ratios haplotype associations patterns may reflect heterogeneous routes to alter common pathways. The above observations have several practical implications in considering next-generation intervention strategies. First, the networks provide a basis for designing combinatorial therapies. Examination of the networks, and their activity states, provides a rational means of determining which combination of genes need to be targeted in order to alter the state of critical nodes. It is also interesting that not all alterations in pathways states influence outcome. This observed difference in effect on outcome, which may reflect the result of natural experiments by the tumor, may also prove important in prioritizing which genes and interactions might be most productively targeted to improve outcome.

## Materials and Methods

### Mapping Entrez Gene to Golden Path

NCBI's Entrez Gene database contains 36470 human records, 25441 of them annotated as protein-coding. For each gene in this set we used a variety of methods to find its location Golden Path genome sequence. Version (hg18) of the genome database contains extensive annotations which we used wherever possible. In some cases we used BLAT to find genomic locations.

The positions of approximately 18,342 (∼54%) genes were annotated directly in Golden Path's refLink and refGene tables. While this is the most straightforward reference, it leaves 18,128 genes unmapped, 6,757 (∼18.5%) of them protein-coding.

In cases where a direct gene annotation was not available, we searched Golden Path's annotations for the locations of associated sequences from a variety of sources, listed below in order of preference:

- mRNA accessions from Entrez Gene's “gene2accession” table
- cross-referenced accessions from the HUGO database
- cross-referenced accessions from the uniSTS database
- primary representative sequence from associated UniGene cluster
- mRNA sequences from associated UniGene cluster
- EST sequences from associated UniGene cluster

Accessions were gathered from each of these sources in turn, and then looked up in various Golden Path annotation tables (all_mrna, stsMap, clonePos, and all_est). A locally-built database of mRNA and refseq BLAT results (assembled by Robert Clifford) was also searched, providing some additional matches. The resulting genomic locations of the search sequences were aggregated, and accepted as the gene's position if the locations fell within a 3 mb region (3 mb being a somewhat arbitrary cutoff based on the largest observed refLink-based gene mapping of approximately 2.3 mb). If a chromosome annotation was available from Entrez Gene, HUGO, or uniSTS, genomic positions were only included if they were on the same chromosome. A known chromosome annotation was required in the case of UniGene mRNA and EST sequence lookups.

In cases where accession annotations were available but the positions were not found, we performed our own BLAT searches. This was necessary for certain classes of accessions which do not appear in the Golden Path database (e.g. the “XM_” series of predicted refseqs). If a chromosome annotation was available for the gene, a BLAT search was run only against that chromosome, otherwise all chromosomes were searched. Results were aggregated and accepted as the gene's position if they fell within a 10 mb or smaller region. This is a less strict requirement than used in the accession-based mapping system, yet it can provide at least a general position, much more specific than a cytogenetic-based coordinate (the only mapping information available for some Entrez Gene entries). If plausible matches were found on multiple chromosomes, the gene mapping was rejected as ambiguous.

BLAT results are annotated with one of four categories of match types, so the annotations may be excluded later if they are considered too broad. The four categories are:

1. A single perfect match for the query sequence was found. The ideal mapping result.
2. More than one perfect match for the query sequence was found.
3. A single near-perfect match (at least 95% but less than 100% identity) was found.
4. Multiple near-perfect matches were found.

Preferential treatment was given to perfect refseq matches in the results – i.e. a perfect BLAT match to a refseq was considered the gene's genomic position, regardless of the presence of other near-perfect matches in the results.

If mapping failed by any of the above methods a few crude methods of last resort were attempted:

1. if a gene was positioned on an NCBI genomic contig sequence(NC_* series accession, via EG's “gene2refseq” table), and a neighboring gene on the same chromosome, arm,
  and band could be found in Golden Path, the relative
  distance between the two genes in the NCBI sequence
  was applied to the Golden Path coordinates to approximate
  its position.
2. If a gene had only a cytogenetic location available, coordinates of Golden Path-mapped genes with the same cytogenetic location were aggregated and a union of their position generated. The resulting mappings are extremely broad but at least point to a general molecular region which may still be useful in some circumstances.

### Mapping BACs to Golden Path

The second dataset to be mapped to Golden Path consisted of the set of BACs used in the CGH arrays from Chin et al [24]. As with the Entrez Gene mapping process, the Golden Path annotation database contains an ideal table for our purposes, “bacEndPairs”, holding the genomic positions of BACs whose end sequences have both been mapped. However, only approximately 39% of the BACs in our set contain an entry in this table. The “fishClones” table provided mappings for an additional 6% of the BACs. For the remainder we used BAC-related annotations as a basis for mapping.

The NCBI clone registry provided a major source of BAC annotations. From it, we extracted BAC-related accession, end sequence, STS and chromosome information. The registry also provided cross-connections to uniSTS, from which we gathered additional related accessions. We searched for the resulting sequences in Golden Path's all_mrna, clonePos, stsMap, and all_ests tables. We also took special note of any matches for BAC end sequences. In addition to the clone registry, we also used annotations from the UCSF 2.0 arrays (data from http://cancer.ucsf.edu/array/analysis/), as well as GenBank records referencing BAC names in the title block. Genome mappings were accepted for the BACs if they were no longer than 500 kb in length, and mappings to ambiguous chromosomes were rejected.

For BACs which could not be found using NCBI clone registry or UCSF array annotations, we attempted a surrogate-based mapping approach. Chin et als [1] CGH array annotations provided rough genomic positions (in megabases) whose coordinates aligned most closely with an older genome build, hg16. For each BAC, we extracted sequence IDs from hg16 which were annotated as being near this position. Sets of sequences were extracted from each of the all_mrna, stsMap, and all_est annotation tables. For mRNAs and STSs, we used sequences located within plus or minus 5 kb of the target location. For ESTs, we took sequences within plus or minus 1 kb of the target position. These extracted sequences were used as surrogates for the BACs, and looked up in hg18, searching (in order of preference) mRNAs, STSs, and ESTs. This approach was used to generate hg18 positions for approximately 8.7% of the BACs.

For BACs that could not be mapped to hg18 using any of the above methods, a second pass was performed to find generate approximate positions based on interpolated neighboring BAC locations. For each BAC, we tried to find flanking BACs with hg18 mappings. We then applied relative offsets to the hg18 positions based on the spacings in the hg16 positions. This was only required for approximately 1.4% of the BACs.

#### BAC preprocessing

Two sets of modified genomic positions are generated for each BAC, which we refer to as expanded and extended coordinates.

Expanded coordinates are an attempt to compensate for the many cases where BAC mapping and end-sequence information is incomplete. They are intended to ensure that all BACs cover a minimum amount of the genome, and that fully-mapped BACs do not crowd out BACs having less complete mapping annotations. This involves expanding mapped BAC coordinates up to approximately 165kb, which is our observation of the median size of BACs where both end sequences have been mapped. Coordinates are not expanded in cases where both end sequences have been mapped, or if existing mapping information spans 100kb or more. If a single end sequence mapping is known, the expansion is made away from the anchored end, otherwise the coordinates are expanded equally in either direction. Collisions during expansion between closely-mapped BACs are detected and resolved by a multi-pass process where the available intervening space is assigned equally between BACs. If expansion in one direction causes a collision with a neighboring BAC, appropriate compensatory expansion is attempted in the other direction, unless that end is fixed by the presence of a known end sequence.

Extended coordinates build upon the expanded mappings by dividing unassigned regions of the genome between neighboring BACs. This provides pseudo-tiling coverage of the genome, allowing any given region to be associated with the most appropriate BAC in the set. Generating extended coordinates requires expanded coordinates to be calculated first, to allow the most equitable assignment of intervening regions.

Expanded and extended coordinates are computed dynamically based on the BAC membership of the CGH array being worked with. While the hg16-based CGH arrays were intended to sample the genome at regular intervals, their computed positions in hg18 are not as neatly spaced. For these purposes the BACs were arranged as we observed them in hg18.

There are cases where BAC coordinates overlap. In cases where a BAC is computed to lie entirely within a larger BAC, the smaller BAC receives the same final coordinates as the larger BAC (it is essentially considered a duplicate). In cases where a BAC partially overlaps with another, the coordinates in the overlap region are left unchanged, and no expansion or extension is performed on the end with the overlap.

### Associating BACs with genes

There are three basic types of intersections between gene and BAC coordinates:

1. The gene's mapping falls entirely within the BAC's mapping.
2. The gene's mapping lies partly within the BAC's mapping and partly outside.
3. The gene's mapping is larger than the BAC's mapping. This can happen for genes with very broad cytogenetically-derived gene mappings.

Gene-to-BAC associations of the first type are trivial to calculate. The latter two cases require some additional steps to determine whether a gene should be associated with a BAC or not. Associations are generally rejected if the length of the BAC mapping is less than one-third the length of the gene mapping. This prevents associations from being formed based on insubstantial overlaps. If the extended set of BAC coordinates is being used, an association is rejected unless at least 50% of the gene's coordinates lie within the BAC's coordinates. Since in extended mode BACs tile the genome completely, this step ensures that genes in border regions will be assigned to one BAC exclusively. Specific associations of BACs and their genes has been previously described in Chin et al. [24].

Identifying Genes in Copy Number Altered Regions. In order to identify the genes in the copy number altered regions it was necessary to translating BACs coordinate used in the comparative genomic hybridization (CGH) assays into genome coordinates. This involved mapping the Entrez Gene database and the CGH BACs to a common coordinate space (Golden Path human genome build hg18), and then overlaying the results. These processes are described in the supplemental material [19].

### Mapping Genes to Pathways

We determined the list of genes used in each pathway in by query of the Pathway Interaction Database [49].

### p-value for a pathway's genomic alterations in a specific sample

Each pathway network has been taken as a set of genes. That is, for each pathway, and according to (2.4), we listed the genes which are members of the pathway.

To determine the probability that a pathway is to be hit by exactly k hits, we first calculate the probability that the pathway is randomly hit 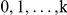 times. With G genes quantified in a given platform (for example, a platform that covers the entire genome will cover roughly G = 24,000), and N_i_ genes in a pathway i (N_i_ is usually between 10–70 genes) we get:
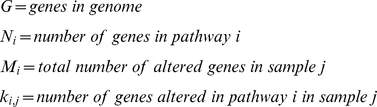
(2.4)The probability of randomly hitting zero to *k_i_*_,*j*_ genes, given that *M_j_* genes are altered in sample *j*is the hypergeometric cumulative distribution function:
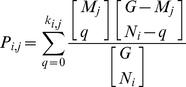
(2.5)The associated p-value is therefore defined as:
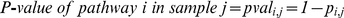
(2.6)


### p-value for a global pathway targeting across a population

To be able to statistically quantify genomic targeting of a pathway across a population of subjects we need to iterate across the p-values defined in (2.5). This is in effect a combination of one sided binomial tests. This has been solved by different techniques, including Fisher's Omnibus [50], which we are using here. This test statistics for pathway *i* is expressed here as:
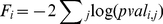
(2.7)and the corresponding p-value is:
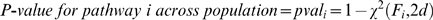
(2.8)where 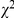 is the Chi-square cumulative distribution function and *d* are the number of degrees of freedom (number of samples).

## Supporting Information

Table S1 Bonferroni correction was applied on the p-values calculated using the Fisher Omnibus test in order to address the problem of multiple comparisons. The value for significance was assign to be 8.834×10^−5^, which is 0.05/566 (when 566 is the number of pathways). Table S1 shows all 566 pathways calculated from Chin's dataset with the p-value calculated via Fisher Omnibus test. In addition, every p-value was adjusted and pathway significance was reassigned.
(0.65 MB DOC)

Table S2 Table S2 shows the entire panel of subjects for the following pathway “cdc25 and chk1 regulatory pathway in response to DNA damage”. This pathway is composed of 9 genes. This table shows the copy number alterations across 145 breast cancer patient: −1 indicates deletion, 1 indicates amplification and 0 indicates of no significant change.
(0.19 MB DOC)

Table S3 Table S3, presented here, shows all pathways that found to be significant using Kaplan-Meier survival analysis. All of the pathways presented here were found to be significantly targeted through copy number alteration using the Fisher Omnibus test (after correction). All 29 pathways were tested in two more public datasets obtain from GEO (http://www.ncbi.nlm.nih.gov/geo). A - activity, C - consistency.
(0.05 MB DOC)

Table S4 The table details the Fisher's Omnibus value for each pathway. Columns 3 and onward give the detailed p-value obtained through the Hypergeometric function, as it has been calculated per patient, per pathway.
(1.56 MB XLS)
