# Supplementary material for: Detecting Cancer Gene Networks Characterized by Recurrent Genomic Alterations in a Population
Source: PLoS One. 2011 Jan 4;6(1):e14437. doi: 10.1371/journal.pone.0014437 (PMC3014942; doi:10.1371/journal.pone.0014437)
Supplement: Table S1 — Bonferroni correction was applied on the p-values calculated using the Fisher Omnibus test in order to address the problem of multiple comparisons. The value for significance was assign to be 8.834×10^−5^, which is 0.05/566 (when 566 is the number of pathways). Table S1 shows all 566 pathways calculated from Chin's dataset with the p-value calculated via Fisher Omnibus test. In addition, every p-value was adjusted and pathway significance was reassigned. (0.65 MB DOC) [file pone.0014437.s001.doc]

| Pathway name | Fisher omnibus P-value | Number of genes in pathway | Pathway significant after Bonferroni correction |
| --- | --- | --- | --- |
| 1- and 2-Methylnaphthalene degradation (Kegg) | 0 | 2 |  |
| 1,4-Dichlorobenzene degradation(Kegg) | 0 | 1 |  |
| a4b1 and a4b7 Integrin signaling(NCI/Nature) | 0 | 2 |  |
| actions of nitric oxide in the heart(BioCarta) | 0 | 14 |  |
| activation of pkc through g-protein coupled receptors(BioCarta) | 0 | 2 |  |
| adp-ribosylation factor(BioCarta) | 0 | 3 |  |
| ahr signal transduction pathway(BioCarta) | 0 | 2 |  |
| Alkaloid biosynthesis I(Kegg) | 0 | 3 |  |
| alpha-Linolenic acid metabolism(Kegg) | 0 | 1 |  |
| alpha-synuclein and parkin-mediated proteolysis in parkinson`s disease(BioCarta) | 0 | 2 |  |
| Aminoacyl-tRNA biosynthesis(Kegg) | 0 | 4 |  |
| Aminophosphonate metabolism(Kegg) | 0 | 1 |  |
| antisense pathway(BioCarta) | 0 | 4 |  |
| Arf6 mediated densensitization of LHCGR(NCI/Nature) | 0 | 1 |  |
| Ascorbate and aldarate metabolism(Kegg) | 0 | 2 |  |
| Asthma(Kegg) | 0 | 2 |  |
| basic mechanism of action of ppara pparb(d) and pparg and effects on gene expression(BioCarta) | 0 | 7 |  |
| Benzoate degradation via CoA ligation(Kegg) | 0 | 1 |  |
| beta-Alanine metabolism(Kegg) | 0 | 11 |  |
| bioactive peptide induced signaling pathway(BioCarta) | 0 | 23 |  |
| Biosynthesis of steroids(Kegg) | 0 | 20 |  |
| Biotin metabolism(Kegg) | 0 | 2 |  |
| btg family proteins and cell cycle regulation(BioCarta) | 0 | 6 |  |
| C5-Branched dibasic acid metabolism(Kegg) | 0 | 2 |  |
| ca-calmodulin-dependent protein kinase activation(BioCarta) | 0 | 2 |  |
| Caffeine metabolism(Kegg) | 0 | 2 |  |
| Calcium signaling in the CD4+ TCR pathway(NCI/Nature) | 0 | 26 |  |
| Calcium signaling pathway(Kegg) | 0 | 1 |  |
| Caprolactam degradation(Kegg) | 0 | 1 |  |
| Carbon fixation(Kegg) | 0 | 3 |  |
| cdk regulation of dna replication(BioCarta) | 0 | 8 |  |
| Cell adhesion molecules (CAMs)(Kegg) | 0 | 68 |  |
| Chondroitin sulfate biosynthesis(Kegg) | 0 | 1 |  |
| Circadian rhythm(Kegg) | 0 | 2 |  |
| Citrate cycle (TCA cycle)(Kegg) | 0 | 8 |  |
| Class IB PI3K non-lipid kinase events(NCI/Nature) | 0 | 3 |  |
| Complement and coagulation cascades(Kegg) | 0 | 44 |  |
| Cyanoamino acid metabolism(Kegg) | 0 | 1 |  |
| Cytokine-cytokine receptor interaction(Kegg) | 0 | 179 |  |
| D-Arginine and D-ornithine metabolism(Kegg) | 0 | 1 |  |
| degradation of the rar and rxr by the proteasome(BioCarta) | 0 | 5 |  |
| deregulation of cdk5 in alzheimers disease(BioCarta) | 0 | 6 |  |
| dicer pathway(BioCarta) | 0 | 1 |  |
| Effects of Botulinum toxin(NCI/Nature) | 0 | 14 |  |
| Ephrin A reverse signaling(NCI/Nature) | 0 | 3 |  |
| EphrinA-EPHA pathway(NCI/Nature) | 0 | 2 |  |
| Ether lipid metabolism(Kegg) | 0 | 4 |  |
| extrinsic prothrombin activation pathway(BioCarta) | 0 | 13 |  |
| Fatty acid biosynthesis(Kegg) | 0 | 2 |  |
| Fatty acid elongation in mitochondria(Kegg) | 0 | 2 |  |
| fibrinolysis pathway(BioCarta) | 0 | 13 |  |
| Fluorobenzoate degradation(Kegg) | 0 | 1 |  |
| gamma-Hexachlorocyclohexane degradation(Kegg) | 0 | 1 |  |
| generation of amyloid b-peptide by ps1(BioCarta) | 0 | 3 |  |
| Glutathione metabolism(Kegg) | 0 | 5 |  |
| Glypican pathway(NCI/Nature) | 0 | 3 |  |
| g-protein signaling through tubby proteins(BioCarta) | 0 | 7 |  |
| Graft-versus-host disease(Kegg) | 0 | 3 |  |
| g-secretase mediated erbb4 signaling pathway(BioCarta) | 0 | 3 |  |
| how does salmonella hijack a cell(BioCarta) | 0 | 6 |  |
| il 6 signaling pathway(BioCarta) | 0 | 8 |  |
| Inositol metabolism(Kegg) | 0 | 2 |  |
| Insulin-mediated glucose transport(NCI/Nature) | 0 | 7 |  |
| ionomycin and phorbal ester signaling pathway(BioCarta) | 0 | 4 |  |
| JNK signaling in the CD4+ TCR pathway(NCI/Nature) | 0 | 7 |  |
| lck and fyn tyrosine kinases in initiation of tcr activation(BioCarta) | 0 | 6 |  |
| Linoleic acid metabolism(Kegg) | 0 | 2 |  |
| Lipoic acid metabolism(Kegg) | 0 | 2 |  |
| LPA4-mediated signaling events(NCI/Nature) | 0 | 3 |  |
| Lysine biosynthesis(Kegg) | 0 | 3 |  |
| mcalpain and friends in cell motility(BioCarta) | 0 | 15 |  |
| mechanism of acetaminophen activity and toxicity(BioCarta) | 0 | 7 |  |
| mechanism of gene regulation by peroxisome proliferators via ppara(BioCarta) | 0 | 7 |  |
| mechanisms of transcriptional repression by dna methylation(BioCarta) | 0 | 3 |  |
| Metabolism of xenobiotics by cytochrome P450(Kegg) | 0 | 2 |  |
| neuroregulin receptor degredation protein-1 controls erbb3 receptor recycling(BioCarta) | 0 | 3 |  |
| Novobiocin biosynthesis(Kegg) | 0 | 1 |  |
| opposing roles of aif in apoptosis and cell survival(BioCarta) | 0 | 2 |  |
| Paxillin-dependent events mediated by a4b1(NCI/Nature) | 0 | 12 |  |
| pelp1 modulation of estrogen receptor activity(BioCarta) | 0 | 7 |  |
| Peptidoglycan biosynthesis(Kegg) | 0 | 1 |  |
| Phenylalanine metabolism(Kegg) | 0 | 4 |  |
| Phenylalanine, tyrosine and tryptophan biosynthesis(Kegg) | 0 | 2 |  |
| phospholipase c delta in phospholipid associated cell signaling(BioCarta) | 0 | 4 |  |
| phospholipase c signaling pathway(BioCarta) | 0 | 3 |  |
| polyadenylation of mrna(BioCarta) | 0 | 4 |  |
| proteasome complex(BioCarta) | 0 | 4 |  |
| pten dependent cell cycle arrest and apoptosis(BioCarta) | 0 | 11 |  |
| Reductive carboxylate cycle (CO2 fixation)(Kegg) | 0 | 3 |  |
| regulation of map kinase pathways through dual specificity phosphatases(BioCarta) | 0 | 1 |  |
| Regulation of nuclear SMAD2/3 signaling(NCI/Nature) | 0 | 56 |  |
| reversal of insulin resistance by leptin(BioCarta) | 0 | 5 |  |
| S1P4 pathway(NCI/Nature) | 0 | 3 |  |
| S1P5 pathway(NCI/Nature) | 0 | 2 |  |
| SNARE interactions in vesicular transport(Kegg) | 0 | 3 |  |
| Sphingosine 1-phosphate (S1P) pathway(NCI/Nature) | 0 | 1 |  |
| srebp control of lipid synthesis(BioCarta) | 0 | 5 |  |
| stat3 signaling pathway(BioCarta) | 0 | 9 |  |
| Streptomycin biosynthesis(Kegg) | 0 | 3 |  |
| stress induction of hsp regulation(BioCarta) | 0 | 18 |  |
| Sulfur metabolism(Kegg) | 0 | 3 |  |
| Synthesis and degradation of ketone bodies(Kegg) | 0 | 1 |  |
| telomeres telomerase cellular aging and immortality(BioCarta) | 0 | 11 |  |
| Terpenoid biosynthesis(Kegg) | 0 | 3 |  |
| Tetrachloroethene degradation(Kegg) | 0 | 1 |  |
| the prc2 complex sets long-term gene silencing through modification of histone tails(BioCarta) | 0 | 2 |  |
| Thiamine metabolism(Kegg) | 0 | 3 |  |
| Thyroid cancer(Kegg) | 0 | 5 |  |
| trka receptor signaling pathway(BioCarta) | 0 | 8 |  |
| Type I diabetes mellitus(Kegg) | 0 | 3 |  |
| Valine, leucine and isoleucine biosynthesis(Kegg) | 0 | 1 |  |
| Vibrio cholerae infection(Kegg) | 0 | 6 |  |
| inhibition of matrix metalloproteinases(BioCarta) | 1.11E-16 | 9 |  |
| il-10 anti-inflammatory signaling pathway(BioCarta) | 2.22E-16 | 11 |  |
| rho-selective guanine exchange factor akap13 mediates stress fiber formation(BioCarta) | 2.22E-16 | 6 |  |
| Role of Calcineurin-dependent NFAT signaling in lymphocytes(NCI/Nature) | 2.22E-16 | 25 |  |
| S1P2 pathway(NCI/Nature) | 2.22E-16 | 5 |  |
| Small cell lung cancer(Kegg) | 2.22E-16 | 10 |  |
| FOXA transcription factor networks(NCI/Nature) | 4.44E-16 | 3 |  |
| Long-term depression(Kegg) | 4.44E-16 | 9 |  |
| Styrene degradation(Kegg) | 5.55E-16 | 3 |  |
| integrin signaling pathway(BioCarta) | 6.66E-16 | 25 |  |
| EPHA2 forward signaling(NCI/Nature) | 7.77E-16 | 13 |  |
| role of nicotinic acetylcholine receptors in the regulation of apoptosis(BioCarta) | 1.67E-15 | 14 |  |
| Riboflavin metabolism(Kegg) | 2.44E-15 | 3 |  |
| acetylation and deacetylation of rela in nucleus(BioCarta) | 2.89E-15 | 3 |  |
| Non-small cell lung cancer(Kegg) | 3.11E-15 | 12 |  |
| phosphatidylcholine biosynthesis pathway(BioCarta) | 3.55E-15 | 3 |  |
| Dentatorubropallidoluysian atrophy (DRPLA)(Kegg) | 5.33E-15 | 6 |  |
| ras-independent pathway in nk cell-mediated cytotoxicity(BioCarta) | 5.55E-15 | 14 |  |
| cbl mediated ligand-induced downregulation of egf receptors pathway(BioCarta) | 5.77E-15 | 7 |  |
| Olfactory transduction(Kegg) | 7.99E-15 | 6 |  |
| y branching of actin filaments(BioCarta) | 9.77E-15 | 10 |  |
| Signaling mediated by p38-gamma and p38-delta(NCI/Nature) | 1.17E-14 | 4 |  |
| a6b1 and a6b4 Integrin signaling(NCI/Nature) | 1.19E-14 | 21 |  |
| Jak-STAT signaling pathway(Kegg) | 1.94E-14 | 7 |  |
| cell to cell adhesion signaling(BioCarta) | 2.00E-14 | 10 |  |
| cystic fibrosis transmembrane conductance regulator (cftr) and beta 2 adrenergic receptor (b2ar) pathway(BioCarta) | 3.11E-14 | 8 |  |
| regulation of splicing through sam68(BioCarta) | 3.34E-14 | 10 |  |
| cardiac protection against ros(BioCarta) | 3.75E-14 | 3 |  |
| ucalpain and friends in cell spread(BioCarta) | 3.80E-14 | 10 |  |
| platelet amyloid precursor protein pathway(BioCarta) | 3.89E-14 | 7 |  |
| Aurora C signaling(NCI/Nature) | 4.92E-14 | 3 |  |
| gata3 participate in activating the th2 cytokine genes expression(BioCarta) | 5.71E-14 | 10 |  |
| Methane metabolism(Kegg) | 1.60E-13 | 3 |  |
| Pathogenic Escherichia coli infection(Kegg) | 1.69E-13 | 4 |  |
| gamma-aminobutyric acid receptor life cycle pathway(BioCarta) | 3.07E-13 | 11 |  |
| Arf6 downstream pathway(NCI/Nature) | 3.26E-13 | 10 |  |
| Pantothenate and CoA biosynthesis(Kegg) | 3.33E-13 | 8 |  |
| GnRH signaling pathway(Kegg) | 3.78E-13 | 16 |  |
| Signaling mediated by p38-alpha and p38-beta(NCI/Nature) | 4.79E-13 | 21 |  |
| angiotensin ii mediated activation of jnk pathway via pyk2 dependent signaling(BioCarta) | 5.07E-13 | 24 |  |
| role of pi3k subunit p85 in regulation of actin organization and cell migration(BioCarta) | 5.35E-13 | 10 |  |
| links between pyk2 and map kinases(BioCarta) | 6.25E-13 | 25 |  |
| phospholipids as signalling intermediaries(BioCarta) | 7.82E-13 | 22 |  |
| IL4-mediated signaling events(NCI/Nature) | 7.94E-13 | 56 |  |
| Adipocytokine signaling pathway(Kegg) | 8.37E-13 | 21 |  |
| Glycerophospholipid metabolism(Kegg) | 8.51E-13 | 16 |  |
| Bladder cancer(Kegg) | 1.25E-12 | 9 |  |
| overview of telomerase rna component gene hterc transcriptional regulation(BioCarta) | 1.52E-12 | 5 |  |
| intrinsic prothrombin activation pathway(BioCarta) | 1.58E-12 | 17 |  |
| roles of ß arrestin dependent recruitment of src kinases in gpcr signaling(BioCarta) | 1.71E-12 | 18 |  |
| antigen processing and presentation(BioCarta) | 2.00E-12 | 6 |  |
| nitric oxide signaling pathway(BioCarta) | 2.06E-12 | 6 |  |
| ß-arrestins in gpcr desensitization(BioCarta) | 2.28E-12 | 10 |  |
| Taurine and hypotaurine metabolism(Kegg) | 2.83E-12 | 3 |  |
| hop pathway in cardiac development(BioCarta) | 4.35E-12 | 5 |  |
| Nitrogen metabolism(Kegg) | 5.26E-12 | 7 |  |
| Natural killer cell mediated cytotoxicity(Kegg) | 5.33E-12 | 42 |  |
| role of erk5 in neuronal survival pathway(BioCarta) | 5.70E-12 | 17 |  |
| Insulin signaling pathway(Kegg) | 1.28E-11 | 29 |  |
| Aminosugars metabolism(Kegg) | 1.30E-11 | 11 |  |
| Noncanonical Wnt signaling pathway(NCI/Nature) | 1.38E-11 | 7 |  |
| ccr3 signaling in eosinophils(BioCarta) | 1.71E-11 | 20 |  |
| thrombin signaling and protease-activated receptors(BioCarta) | 2.05E-11 | 13 |  |
| role of erbb2 in signal transduction and oncology(BioCarta) | 2.27E-11 | 19 |  |
| signal dependent regulation of myogenesis by corepressor mitr(BioCarta) | 2.41E-11 | 5 |  |
| d4gdi signaling pathway(BioCarta) | 3.01E-11 | 7 |  |
| downregulated of mta-3 in er-negative breast tumors(BioCarta) | 3.03E-11 | 15 |  |
| Parkinson's disease(Kegg) | 3.26E-11 | 8 |  |
| VEGF signaling pathway(Kegg) | 3.29E-11 | 14 |  |
| Long-term potentiation(Kegg) | 3.51E-11 | 6 |  |
| internal ribosome entry pathway(BioCarta) | 4.04E-11 | 6 |  |
| activation of camp-dependent protein kinase pka(BioCarta) | 4.49E-11 | 4 |  |
| Methionine metabolism(Kegg) | 4.62E-11 | 9 |  |
| yaci and bcma stimulation of b cell immune responses(BioCarta) | 5.31E-11 | 11 |  |
| Regulation of p38-alpha and p38-beta(NCI/Nature) | 7.79E-11 | 7 |  |
| fmlp induced chemokine gene expression in hmc-1 cells(BioCarta) | 8.95E-11 | 20 |  |
| e2f1 destruction pathway(BioCarta) | 1.11E-10 | 9 |  |
| prion pathway(BioCarta) | 1.15E-10 | 9 |  |
| sprouty regulation of tyrosine kinase signals(BioCarta) | 1.20E-10 | 14 |  |
| ctcf: first multivalent nuclear factor(BioCarta) | 1.48E-10 | 19 |  |
| Melanogenesis(Kegg) | 1.54E-10 | 14 |  |
| Leukocyte transendothelial migration(Kegg) | 1.61E-10 | 28 |  |
| Selenoamino acid metabolism(Kegg) | 2.50E-10 | 3 |  |
| Amyotrophic lateral sclerosis (ALS)(Kegg) | 2.82E-10 | 8 |  |
| Epithelial cell signaling in Helicobacter pylori infection(Kegg) | 2.83E-10 | 19 |  |
| visceral fat deposits and the metabolic syndrome(BioCarta) | 2.89E-10 | 12 |  |
| Alternative NF-kappaB pathway(NCI/Nature) | 3.13E-10 | 4 |  |
| Lysine degradation(Kegg) | 4.08E-10 | 8 |  |
| Nongenotropic Androgen signaling(NCI/Nature) | 4.78E-10 | 16 |  |
| the igf-1 receptor and longevity(BioCarta) | 5.55E-10 | 9 |  |
| t cell receptor signaling pathway(BioCarta) | 7.30E-10 | 25 |  |
| Syndecan-3-mediated signaling events(NCI/Nature) | 7.31E-10 | 11 |  |
| hiv-1 defeats host-mediated resistance by cem15(BioCarta) | 1.05E-09 | 4 |  |
| erk1/erk2 mapk signaling pathway(BioCarta) | 1.28E-09 | 18 |  |
| Systemic lupus erythematosus(Kegg) | 1.30E-09 | 4 |  |
| Syndecan-2-mediated signaling events(NCI/Nature) | 1.33E-09 | 17 |  |
| il 3 signaling pathway(BioCarta) | 1.34E-09 | 9 |  |
| induction of apoptosis through dr3 and dr4/5 death receptors(BioCarta) | 1.39E-09 | 19 |  |
| B cell receptor signaling pathway(Kegg) | 1.41E-09 | 16 |  |
| transcriptional activation of dbpb from mrna(BioCarta) | 1.47E-09 | 6 |  |
| rho cell motility signaling pathway(BioCarta) | 1.62E-09 | 9 |  |
| Ras signaling in the CD4+ TCR pathway(NCI/Nature) | 1.62E-09 | 5 |  |
| Allograft rejection(Kegg) | 1.80E-09 | 5 |  |
| Glycosphingolipid biosynthesis - globoseries(Kegg) | 1.94E-09 | 7 |  |
| role of ß-arrestins in the activation and targeting of map kinases(BioCarta) | 2.32E-09 | 14 |  |
| Endometrial cancer(Kegg) | 2.58E-09 | 12 |  |
| chrebp regulation by carbohydrates and camp(BioCarta) | 2.61E-09 | 8 |  |
| O-Glycan biosynthesis(Kegg) | 2.73E-09 | 5 |  |
| Nectin adhesion pathway(NCI/Nature) | 3.31E-09 | 12 |  |
| chromatin remodeling by hswi/snf atp-dependent complexes(BioCarta) | 3.41E-09 | 7 |  |
| Glyoxylate and dicarboxylate metabolism(Kegg) | 3.63E-09 | 5 |  |
| cycling of ran in nucleocytoplasmic transport(BioCarta) | 4.84E-09 | 9 |  |
| il22 soluble receptor signaling pathway(BioCarta) | 5.17E-09 | 8 |  |
| phosphorylation of mek1 by cdk5/p35 down regulates the map kinase pathway(BioCarta) | 5.42E-09 | 12 |  |
| Retinol metabolism(Kegg) | 5.77E-09 | 6 |  |
| Arf6 trafficking events(NCI/Nature) | 7.90E-09 | 16 |  |
| regulation of p27 phosphorylation during cell cycle progression(BioCarta) | 8.13E-09 | 11 |  |
| Histidine metabolism(Kegg) | 8.62E-09 | 8 |  |
| cyclin e destruction pathway(BioCarta) | 9.22E-09 | 9 |  |
| Signaling events mediated by HDAC Class III(NCI/Nature) | 9.46E-09 | 22 |  |
| Arachidonic acid metabolism(Kegg) | 1.08E-08 | 13 |  |
| Glycosaminoglycan degradation(Kegg) | 1.09E-08 | 10 |  |
| spliceosomal assembly(BioCarta) | 1.43E-08 | 4 |  |
| how progesterone initiates the oocyte maturation(BioCarta) | 1.56E-08 | 17 |  |
| Glycosphingolipid biosynthesis - lactoseries(Kegg) | 1.65E-08 | 8 |  |
| p38 signaling mediated by MAPKAP kinases(NCI/Nature) | 1.72E-08 | 17 |  |
| melanocyte development and pigmentation pathway(BioCarta) | 1.84E-08 | 15 |  |
| alternative complement pathway(BioCarta) | 1.96E-08 | 5 |  |
| Glycolysis / Gluconeogenesis(Kegg) | 2.04E-08 | 8 |  |
| Fc epsilon RI signaling pathway(Kegg) | 2.12E-08 | 12 |  |
| Porphyrin and chlorophyll metabolism(Kegg) | 2.23E-08 | 12 |  |
| Butanoate metabolism(Kegg) | 2.32E-08 | 8 |  |
| estrogen responsive protein efp controls cell cycle and breast tumors growth(BioCarta) | 2.41E-08 | 9 |  |
| epo signaling pathway(BioCarta) | 2.48E-08 | 9 |  |
| IL6-mediated signaling events(NCI/Nature) | 2.92E-08 | 38 |  |
| Wnt signaling(NCI/Nature) | 2.94E-08 | 6 |  |
| Ubiquinone biosynthesis(Kegg) | 3.04E-08 | 5 |  |
| carm1 and regulation of the estrogen receptor(BioCarta) | 3.14E-08 | 11 |  |
| VEGFR3 signaling in lymphatic endothelium(NCI/Nature) | 3.25E-08 | 11 |  |
| map kinase inactivation of smrt corepressor(BioCarta) | 3.26E-08 | 12 |  |
| attenuation of gpcr signaling(BioCarta) | 3.34E-08 | 5 |  |
| IFN-gamma pathway(NCI/Nature) | 3.82E-08 | 29 |  |
| Sphingolipid metabolism(Kegg) | 4.21E-08 | 10 |  |
| role of mef2d in t-cell apoptosis(BioCarta) | 5.42E-08 | 18 |  |
| multi-drug resistance factors(BioCarta) | 5.74E-08 | 6 |  |
| Pyruvate metabolism(Kegg) | 5.97E-08 | 9 |  |
| akap95 role in mitosis and chromosome dynamics(BioCarta) | 6.08E-08 | 6 |  |
| Arf1 pathway(NCI/Nature) | 6.41E-08 | 17 |  |
| calcium signaling by hbx of hepatitis b virus(BioCarta) | 6.48E-08 | 11 |  |
| activation of csk by camp-dependent protein kinase inhibits signaling through the t cell receptor(BioCarta) | 6.51E-08 | 10 |  |
| mechanism of protein import into the nucleus(BioCarta) | 7.62E-08 | 14 |  |
| Trk receptor signaling mediated by the MAPK pathway(NCI/Nature) | 7.65E-08 | 16 |  |
| Nicotinate and nicotinamide metabolism(Kegg) | 8.01E-08 | 6 |  |
| Cellular roles of Anthrax toxin(NCI/Nature) | 8.20E-08 | 6 |  |
| vegf hypoxia and angiogenesis(BioCarta) | 9.35E-08 | 16 |  |
| regulation of eif-4e and p70s6 kinase(BioCarta) | 1.16E-07 | 19 |  |
| ion channels and their functional role in vascular endothelium(BioCarta) | 1.17E-07 | 10 |  |
| role of brca1 brca2 and atr in cancer susceptibility(BioCarta) | 1.28E-07 | 18 |  |
| Regulation of autophagy(Kegg) | 1.32E-07 | 5 |  |
| Adherens junction(Kegg) | 1.36E-07 | 34 |  |
| inhibition of cellular proliferation by gleevec(BioCarta) | 1.38E-07 | 17 |  |
| Hypoxic and oxygen homeostasis regulation of HIF-1-alpha(NCI/Nature) | 1.48E-07 | 9 |  |
| Vitamin B6 metabolism(Kegg) | 1.61E-07 | 5 |  |
| Chronic myeloid leukemia(Kegg) | 1.62E-07 | 15 |  |
| tsp-1 induced apoptosis in microvascular endothelial cell(BioCarta) | 1.77E-07 | 8 |  |
| Alzheimer's disease(Kegg) | 1.91E-07 | 9 |  |
| transcription factor creb and its extracellular signals(BioCarta) | 2.04E-07 | 9 |  |
| PDGFR-beta signaling pathway(NCI/Nature) | 2.14E-07 | 37 |  |
| p53 signaling pathway(Kegg) | 2.15E-07 | 41 |  |
| cdc25 and chk1 regulatory pathway in response to dna damage(BioCarta) | 2.22E-07 | 9 |  |
| overview of telomerase protein component gene htert transcriptional regulation(BioCarta) | 2.46E-07 | 10 |  |
| rac1 cell motility signaling pathway(BioCarta) | 3.24E-07 | 14 |  |
| Paxillin-independent events mediated by a4b1 and a4b7(NCI/Nature) | 3.36E-07 | 21 |  |
| sodd/tnfr1 signaling pathway(BioCarta) | 3.62E-07 | 11 |  |
| Arf6 signaling events(NCI/Nature) | 4.04E-07 | 18 |  |
| p38 MAPK signaling pathway(NCI/Nature) | 4.71E-07 | 10 |  |
| hemoglobins chaperone(BioCarta) | 4.75E-07 | 11 |  |
| egf signaling pathway(BioCarta) | 6.20E-07 | 16 |  |
| Aurora A signaling(NCI/Nature) | 6.35E-07 | 24 |  |
| S1P1 pathway(NCI/Nature) | 7.29E-07 | 12 |  |
| Type II diabetes mellitus(Kegg) | 9.37E-07 | 14 |  |
| EphrinB-EPHB pathway(NCI/Nature) | 1.19E-06 | 4 |  |
| TRAIL signaling pathway(NCI/Nature) | 1.30E-06 | 8 |  |
| Fructose and mannose metabolism(Kegg) | 1.50E-06 | 9 |  |
| multiple antiapoptotic pathways from igf-1r signaling lead to bad phosphorylation(BioCarta) | 1.56E-06 | 7 |  |
| erythropoietin mediated neuroprotection through nf-kb(BioCarta) | 1.57E-06 | 12 |  |
| pkc-catalyzed phosphorylation of inhibitory phosphoprotein of myosin phosphatase(BioCarta) | 1.59E-06 | 13 |  |
| human cytomegalovirus and map kinase pathways(BioCarta) | 1.65E-06 | 13 |  |
| nfat and hypertrophy of the heart (BioCarta) | 1.72E-06 | 29 |  |
| Taste transduction(Kegg) | 1.73E-06 | 7 |  |
| agrin in postsynaptic differentiation(BioCarta) | 1.88E-06 | 13 |  |
| role of ppar-gamma coactivators in obesity and thermogenesis(BioCarta) | 1.92E-06 | 9 |  |
| brca1 dependent ub ligase activity(BioCarta) | 2.07E-06 | 10 |  |
| akt signaling pathway(BioCarta) | 2.08E-06 | 12 |  |
| caspase cascade in apoptosis(BioCarta) | 2.13E-06 | 15 |  |
| sumoylation as a mechanism to modulate ctbp-dependent gene responses(BioCarta) | 2.38E-06 | 10 |  |
| Notch signaling pathway(Kegg) | 2.39E-06 | 4 |  |
| aspirin blocks signaling pathway involved in platelet activation(BioCarta) | 2.45E-06 | 15 |  |
| Class I PI3K signaling events(NCI/Nature) | 2.79E-06 | 22 |  |
| Wnt signaling pathway(Kegg) | 3.22E-06 | 24 |  |
| regulation of pgc-1a(BioCarta) | 3.41E-06 | 13 |  |
| Autoimmune thyroid disease(Kegg) | 3.45E-06 | 6 |  |
| tnfr1 signaling pathway(BioCarta) | 3.48E-06 | 17 |  |
| regulators of bone mineralization(BioCarta) | 3.57E-06 | 8 |  |
| TCR signaling in naïve CD8+ T cells(NCI/Nature) | 3.94E-06 | 31 |  |
| fosb gene expression and drug abuse(BioCarta) | 4.30E-06 | 9 |  |
| effects of calcineurin in keratinocyte differentiation(BioCarta) | 4.70E-06 | 9 |  |
| growth hormone signaling pathway(BioCarta) | 4.74E-06 | 14 |  |
| fc epsilon receptor i signaling in mast cells(BioCarta) | 4.89E-06 | 24 |  |
| p53 signaling pathway(BioCarta) | 5.42E-06 | 13 |  |
| Antigen processing and presentation(Kegg) | 6.08E-06 | 7 |  |
| Melanoma(Kegg) | 7.04E-06 | 9 |  |
| insulin signaling pathway(BioCarta) | 7.81E-06 | 8 |  |
| mTOR signaling pathway(Kegg) | 7.96E-06 | 11 |  |
| Gap junction(Kegg) | 1.08E-05 | 14 |  |
| Galactose metabolism(Kegg) | 1.17E-05 | 7 |  |
| Fatty acid metabolism(Kegg) | 1.30E-05 | 7 |  |
| Cysteine metabolism(Kegg) | 1.32E-05 | 4 |  |
| TCR signaling in naïve CD4+ T cells(NCI/Nature) | 1.41E-05 | 44 |  |
| EPHA forward signaling(NCI/Nature) | 1.55E-05 | 17 |  |
| Colorectal cancer(Kegg) | 1.72E-05 | 12 |  |
| role of parkin in ubiquitin-proteasomal pathway(BioCarta) | 1.75E-05 | 8 |  |
| cadmium induces dna synthesis and proliferation in macrophages(BioCarta) | 1.81E-05 | 16 |  |
| Drug metabolism - cytochrome P450(Kegg) | 1.84E-05 | 5 |  |
| role of ran in mitotic spindle regulation(BioCarta) | 1.87E-05 | 12 |  |
| Propanoate metabolism(Kegg) | 1.89E-05 | 8 |  |
| role of egf receptor transactivation by gpcrs in cardiac hypertrophy(BioCarta) | 2.58E-05 | 26 |  |
| Glypican 1 network(NCI/Nature) | 2.70E-05 | 18 |  |
| role of mitochondria in apoptotic signaling(BioCarta) | 2.71E-05 | 13 |  |
| nerve growth factor pathway (ngf)(BioCarta) | 2.73E-05 | 13 |  |
| erk and pi-3 kinase are necessary for collagen binding in corneal epithelia(BioCarta) | 2.80E-05 | 23 |  |
| trefoil factors initiate mucosal healing(BioCarta) | 3.19E-05 | 27 |  |
| Glypican 2 network(NCI/Nature) | 3.45E-05 | 4 |  |
| the 41bb-dependent immune response(BioCarta) | 3.76E-05 | 11 |  |
| FOXM1 transcription factor network(NCI/Nature) | 3.94E-05 | 19 |  |
| mets affect on macrophage differentiation(BioCarta) | 4.28E-05 | 17 |  |
| regulation of ck1/cdk5 by type 1 glutamate receptors(BioCarta) | 5.26E-05 | 15 |  |
| Acute myeloid leukemia(Kegg) | 5.48E-05 | 17 |  |
| Glycosphingolipid biosynthesis - neo-lactoseries(Kegg) | 7.31E-05 | 12 |  |
| phosphoinositides and their downstream targets(BioCarta) | 7.95E-05 | 18 |  |
| Pentose phosphate pathway(Kegg) | 8.40E-05 | 9 |  |
| S1P3 pathway(NCI/Nature) | 8.61E-05 | 12 |  |
| regulation of bad phosphorylation(BioCarta) | 0.000100052 | 16 |  |
| EPHB forward signaling(NCI/Nature) | 0.000112054 | 23 |  |
| Renal cell carcinoma(Kegg) | 0.000112894 | 13 |  |
| Plasma membrane estrogen receptor signaling(NCI/Nature) | 0.000117557 | 15 |  |
| Huntington's disease(Kegg) | 0.000118682 | 14 |  |
| amb2 Integrin signaling(NCI/Nature) | 0.000122546 | 18 |  |
| FOXA1 transcription factor network(NCI/Nature) | 0.00012317 | 24 |  |
| pdgf signaling pathway(BioCarta) | 0.000128334 | 22 |  |
| Glioma(Kegg) | 0.0001445 | 11 |  |
| Signaling events mediated by the Hedgehog family(NCI/Nature) | 0.000170724 | 21 |  |
| lissencephaly gene (lis1) in neuronal migration and development(BioCarta) | 0.000222732 | 11 |  |
| p38 mapk signaling pathway(BioCarta) | 0.00023259 | 24 |  |
| Focal adhesion(Kegg) | 0.000257473 | 30 |  |
| il 4 signaling pathway(BioCarta) | 0.000277238 | 19 |  |
| repression of pain sensation by the transcriptional regulator dream(BioCarta) | 0.000282155 | 10 |  |
| RXR and RAR heterodimerization with other nuclear receptor(NCI/Nature) | 0.000315978 | 12 |  |
| proteolysis and signaling pathway of notch(BioCarta) | 0.000347452 | 8 |  |
| influence of ras and rho proteins on g1 to s transition(BioCarta) | 0.000380726 | 25 |  |
| One carbon pool by folate(Kegg) | 0.000565966 | 11 |  |
| atm signaling pathway(BioCarta) | 0.000599546 | 19 |  |
| regulation of cell cycle progression by plk3(BioCarta) | 0.000676763 | 18 |  |
| Glutamate metabolism(Kegg) | 0.000694067 | 15 |  |
| Folate biosynthesis(Kegg) | 0.000710863 | 7 |  |
| no2-dependent il-12 pathway in nk cells(BioCarta) | 0.000743935 | 5 |  |
| basic mechanisms of sumoylation(BioCarta) | 0.000776609 | 9 |  |
| FAS signaling pathway (CD95)(NCI/Nature) | 0.000786588 | 19 |  |
| Alanine and aspartate metabolism(Kegg) | 0.000793281 | 19 |  |
| tumor suppressor arf inhibits ribosomal biogenesis(BioCarta) | 0.000799238 | 19 |  |
| cxcr4 signaling pathway(BioCarta) | 0.000799402 | 8 |  |
| Osteopontin-mediated events(NCI/Nature) | 0.000834036 | 15 |  |
| tgf beta signaling pathway(BioCarta) | 0.000941193 | 20 |  |
| Neuroactive ligand-receptor interaction(Kegg) | 0.000985607 | 8 |  |
| Inositol phosphate metabolism(Kegg) | 0.00111284 | 8 |  |
| Androgen and estrogen metabolism(Kegg) | 0.001264333 | 6 |  |
| Maturity onset diabetes of the young(Kegg) | 0.001288838 | 21 |  |
| keratinocyte differentiation(BioCarta) | 0.001334384 | 41 |  |
| tpo signaling pathway(BioCarta) | 0.001424344 | 19 |  |
| il-7 signal transduction(BioCarta) | 0.001449965 | 14 |  |
| Prostate cancer(Kegg) | 0.001481218 | 16 |  |
| classical complement pathway(BioCarta) | 0.00150487 | 17 |  |
| Signaling events mediated by PTP1B(NCI/Nature) | 0.001582886 | 33 |  |
| Fc-epsilon receptor I signaling in mast cells(NCI/Nature) | 0.001646859 | 31 |  |
| control of gene expression by vitamin d receptor(BioCarta) | 0.001663462 | 17 |  |
| Canonical NF-kappaB pathway(NCI/Nature) | 0.001727135 | 17 |  |
| signaling pathway from g-protein families(BioCarta) | 0.001733949 | 12 |  |
| Visual signal transduction: Cones(NCI/Nature) | 0.002034687 | 24 |  |
| il-2 receptor beta chain in t cell activation(BioCarta) | 0.002091669 | 38 |  |
| FOXA2 and FOXA3 transcription factor networks(NCI/Nature) | 0.002144296 | 23 |  |
| rb tumor suppressor/checkpoint signaling in response to dna damage(BioCarta) | 0.002147439 | 15 |  |
| C21-Steroid hormone metabolism(Kegg) | 0.002161127 | 5 |  |
| Glycosylphosphatidylinositol(GPI)-anchor biosynthesis(Kegg) | 0.002283702 | 17 |  |
| ifn gamma signaling pathway(BioCarta) | 0.002531653 | 9 |  |
| corticosteroids and cardioprotection(BioCarta) | 0.003100114 | 16 |  |
| bcr signaling pathway(BioCarta) | 0.003101271 | 29 |  |
| Drug metabolism - other enzymes(Kegg) | 0.003112835 | 13 |  |
| LPA receptor mediated events(NCI/Nature) | 0.003424128 | 29 |  |
| Bile acid biosynthesis(Kegg) | 0.003607491 | 6 |  |
| Downstream signaling in naïve CD8+ T cells(NCI/Nature) | 0.003742669 | 19 |  |
| fas signaling pathway (cd95)(BioCarta) | 0.003834979 | 18 |  |
| FoxO family signaling(NCI/Nature) | 0.003873467 | 18 |  |
| IL2 signaling events mediated by STAT5(NCI/Nature) | 0.004052069 | 21 |  |
| eukaryotic protein translation(BioCarta) | 0.004083665 | 9 |  |
| ifn alpha signaling pathway(BioCarta) | 0.004516047 | 14 |  |
| T cell receptor signaling pathway(Kegg) | 0.004851938 | 22 |  |
| Nucleotide sugars metabolism(Kegg) | 0.004956286 | 6 |  |
| Signaling events regulated by Ret tyrosine kinase(NCI/Nature) | 0.005076633 | 32 |  |
| cell cycle: g2/m checkpoint(BioCarta) | 0.005273917 | 25 |  |
| apoptotic dna-fragmentation and tissue homeostasis(BioCarta) | 0.005512735 | 5 |  |
| IGF1 pathway(NCI/Nature) | 0.005544636 | 21 |  |
| Biosynthesis of unsaturated fatty acids(Kegg) | 0.0059659 | 5 |  |
| Angiopoietin receptor Tie2-mediated signaling(NCI/Nature) | 0.006171706 | 23 |  |
| pertussis toxin-insensitive ccr5 signaling in macrophage(BioCarta) | 0.006206145 | 7 |  |
| metabolism of anandamide an endogenous cannabinoid(BioCarta) | 0.006731346 | 8 |  |
| skeletal muscle hypertrophy is regulated via akt-mtor pathway(BioCarta) | 0.007420632 | 22 |  |
| PDGFR-alpha signaling pathway(NCI/Nature) | 0.007572257 | 18 |  |
| Endothelins(NCI/Nature) | 0.008187447 | 18 |  |
| anthrax toxin mechanism of action(BioCarta) | 0.008710551 | 7 |  |
| the information processing pathway at the ifn beta enhancer(BioCarta) | 0.008879598 | 13 |  |
| hypoxia-inducible factor in the cardivascular system(BioCarta) | 0.008937864 | 15 |  |
| inhibition of huntingtons disease neurodegeneration by histone deacetylase inhibitors(BioCarta) | 0.009348396 | 6 |  |
| Basal cell carcinoma(Kegg) | 0.009972502 | 5 |  |
| Tyrosine metabolism(Kegg) | 0.010709296 | 17 |  |
| wnt lrp6 signalling(BioCarta) | 0.012764651 | 7 |  |
| control of skeletal myogenesis by hdac and calcium/calmodulin-dependent kinase (camk)(BioCarta) | 0.015086978 | 14 |  |
| ras signaling pathway(BioCarta) | 0.016985181 | 16 |  |
| transcription regulation by methyltransferase of carm1(BioCarta) | 0.017116282 | 9 |  |
| endocytotic role of ndk phosphins and dynamin(BioCarta) | 0.017148097 | 9 |  |
| Glycine, serine and threonine metabolism(Kegg) | 0.017519863 | 23 |  |
| Neurotrophic factor-mediated Trk receptor signaling(NCI/Nature) | 0.018552504 | 36 |  |
| Urea cycle and metabolism of amino groups(Kegg) | 0.018993186 | 16 |  |
| Regulation of actin cytoskeleton(Kegg) | 0.019646384 | 16 |  |
| Pyrimidine metabolism(Kegg) | 0.024066924 | 18 |  |
| bone remodeling(BioCarta) | 0.024083967 | 18 |  |
| Syndecan-1-mediated signaling events(NCI/Nature) | 0.024899277 | 16 |  |
| Syndecan-4-mediated signaling events(NCI/Nature) | 0.024899429 | 25 |  |
| nfkb activation by nontypeable hemophilus influenzae(BioCarta) | 0.024931638 | 27 |  |
| regulation of spermatogenesis by crem(BioCarta) | 0.025991602 | 11 |  |
| Regulation of cytoplasmic and nuclear SMAD2/3 signaling(NCI/Nature) | 0.02601414 | 10 |  |
| nuclear receptors coordinate the activities of chromatin remodeling complexes and coactivators to facilitate initiation of transcription in carcinoma cells(BioCarta) | 0.026144256 | 15 |  |
| chaperones modulate interferon signaling pathway(BioCarta) | 0.026857151 | 15 |  |
| Coregulation of Androgen receptor activity(NCI/Nature) | 0.027364329 | 28 |  |
| granzyme a mediated apoptosis pathway(BioCarta) | 0.027540108 | 13 |  |
| tnf/stress related signaling(BioCarta) | 0.027599976 | 18 |  |
| mtor signaling pathway(BioCarta) | 0.027827281 | 27 |  |
| alk in cardiac myocytes(BioCarta) | 0.028593777 | 14 |  |
| Regulation of Androgen receptor activity(NCI/Nature) | 0.028886275 | 19 |  |
| IL12 signaling mediated by STAT4(NCI/Nature) | 0.029897725 | 14 |  |
| Insulin Pathway(NCI/Nature) | 0.030612223 | 23 |  |
| Glycerolipid metabolism(Kegg) | 0.031596467 | 9 |  |
| Calcineurin-regulated NFAT-dependent transcription in lymphocytes(NCI/Nature) | 0.033480161 | 32 |  |
| N-Glycan biosynthesis(Kegg) | 0.036637999 | 24 |  |
| Glypican 3 network(NCI/Nature) | 0.037154652 | 10 |  |
| Integrins in angiogenesis(NCI/Nature) | 0.037641586 | 32 |  |
| IL23-mediated signaling events(NCI/Nature) | 0.039576941 | 29 |  |
| nf-kb signaling pathway(BioCarta) | 0.03997853 | 15 |  |
| TGF-beta signaling pathway(Kegg) | 0.040492604 | 15 |  |
| igf-1 signaling pathway(BioCarta) | 0.043873458 | 18 |  |
| il 2 signaling pathway(BioCarta) | 0.043888585 | 12 |  |
| eicosanoid metabolism(BioCarta) | 0.045496405 | 28 |  |
| ErbB signaling pathway(Kegg) | 0.048591882 | 27 |  |
| BARD1 signaling events(NCI/Nature) | 0.051540646 | 24 |  |
| Ceramide signaling pathway(NCI/Nature) | 0.053749287 | 35 |  |
| TNF receptor signaling pathway(NCI/Nature) | 0.055820584 | 20 |  |
| Class I PI3K signaling events mediated by Akt(NCI/Nature) | 0.059513895 | 15 |  |
| Visual signal transduction: Rods(NCI/Nature) | 0.062648193 | 21 |  |
| ceramide signaling pathway(BioCarta) | 0.062928679 | 32 |  |
| Thromboxane A2 receptor signaling(NCI/Nature) | 0.074807747 | 22 |  |
| Ephrin B reverse signaling(NCI/Nature) | 0.080308549 | 13 |  |
| PPAR signaling pathway(Kegg) | 0.089065122 | 40 |  |
| Signaling events activated by Hepatocyte Growth Factor Receptor (c-Met)(NCI/Nature) | 0.092441965 | 40 |  |
| IL2 signaling events mediated by PI3K(NCI/Nature) | 0.092663829 | 28 |  |
| protein kinase a at the centrosome(BioCarta) | 0.098458699 | 10 |  |
| toll-like receptor pathway(BioCarta) | 0.104397419 | 33 |  |
| the co-stimulatory signal during t-cell activation(BioCarta) | 0.105353854 | 12 |  |
| Atypical NF-kappaB pathway(NCI/Nature) | 0.10733308 | 13 |  |
| cd40l signaling pathway(BioCarta) | 0.111995029 | 13 |  |
| double stranded rna induced gene expression(BioCarta) | 0.112954414 | 11 |  |
| Signaling events mediated by PRL(NCI/Nature) | 0.1156791 | 12 |  |
| role of mal in rho-mediated activation of srf(BioCarta) | 0.120446347 | 21 |  |
| Aurora B signaling(NCI/Nature) | 0.127663748 | 21 |  |
| MAPK signaling pathway(Kegg) | 0.13739981 | 75 |  |
| Signaling events mediated by VEGFR1 and VEGFR2(NCI/Nature) | 0.148064855 | 46 |  |
| hypoxia and p53 in the cardiovascular system(BioCarta) | 0.155876029 | 21 |  |
| stathmin and breast cancer resistance to antimicrotubule agents(BioCarta) | 0.1575789 | 16 |  |
| phospholipase c-epsilon pathway(BioCarta) | 0.161411261 | 12 |  |
| Lissencephaly gene (LIS1) in neuronal migration and development(NCI/Nature) | 0.165205525 | 34 |  |
| west nile virus(BioCarta) | 0.179133339 | 10 |  |
| b cell survival pathway(BioCarta) | 0.186771747 | 10 |  |
| sonic hedgehog receptor ptc1 regulates cell cycle(BioCarta) | 0.196072638 | 8 |  |
| lectin induced complement pathway(BioCarta) | 0.20202387 | 18 |  |
| HIV-1 Nef: Negative effector of Fas and TNF-alpha(NCI/Nature) | 0.202420741 | 24 |  |
| Hedgehog signaling events mediated by Gli proteins(NCI/Nature) | 0.233311279 | 26 |  |
| p75(NTR)-mediated signaling(NCI/Nature) | 0.236106449 | 26 |  |
| VEGFR1 specific signals(NCI/Nature) | 0.249683982 | 20 |  |
| visual signal transduction(BioCarta) | 0.265287781 | 16 |  |
| Arginine and proline metabolism(Kegg) | 0.270855862 | 10 |  |
| mapkinase signaling pathway(BioCarta) | 0.28763276 | 52 |  |
| ECM-receptor interaction(Kegg) | 0.308542201 | 12 |  |
| Regulation of Telomerase(NCI/Nature) | 0.30999133 | 43 |  |
| Caspase cascade in apoptosis(NCI/Nature) | 0.314961661 | 20 |  |
| signal transduction through il1r(BioCarta) | 0.331356258 | 25 |  |
| Pentose and glucuronate interconversions(Kegg) | 0.334860816 | 7 |  |
| TGF-beta receptor signaling(NCI/Nature) | 0.38910972 | 40 |  |
| BCR signaling pathway(NCI/Nature) | 0.399818142 | 47 |  |
| regulation of transcriptional activity by pml(BioCarta) | 0.4069398 | 14 |  |
| Glycosphingolipid biosynthesis - ganglioseries(Kegg) | 0.41331884 | 8 |  |
| Reelin signaling pathway(NCI/Nature) | 0.428544551 | 29 |  |
| IL1-mediated signaling events(NCI/Nature) | 0.431715285 | 23 |  |
| il12 and stat4 dependent signaling pathway in th1 development(BioCarta) | 0.446695727 | 13 |  |
| tnfr2 signaling pathway(BioCarta) | 0.448695055 | 15 |  |
| Trk receptor signaling mediated by PI3K and PLC-gamma(NCI/Nature) | 0.524286539 | 18 |  |
| FGF signaling pathway(NCI/Nature) | 0.52672564 | 34 |  |
| Hedgehog signaling pathway(Kegg) | 0.529933821 | 8 |  |
| Pancreatic cancer(Kegg) | 0.534956545 | 16 |  |
| apoptotic signaling in response to dna damage(BioCarta) | 0.53515215 | 13 |  |
| Retinoic acid receptors-mediated signaling(NCI/Nature) | 0.544641477 | 17 |  |
| Purine metabolism(Kegg) | 0.5517192 | 25 |  |
| rna polymerase iii transcription(BioCarta) | 0.572536307 | 10 |  |
| IL2-mediated signaling events(NCI/Nature) | 0.575991452 | 34 |  |
| BMP receptor signaling(NCI/Nature) | 0.576986409 | 29 |  |
| Tight junction(Kegg) | 0.595908614 | 21 |  |
| Tryptophan metabolism(Kegg) | 0.603251482 | 15 |  |
| HIF-1-alpha transcription factor network(NCI/Nature) | 0.603786803 | 47 |  |
| Axon guidance(Kegg) | 0.634320618 | 32 |  |
| Starch and sucrose metabolism(Kegg) | 0.644894261 | 11 |  |
| cyclins and cell cycle regulation(BioCarta) | 0.648793501 | 19 |  |
| Signaling events mediated by Stem cell factor receptor (c-Kit)(NCI/Nature) | 0.65194593 | 42 |  |
| oxidative stress induced gene expression via nrf2(BioCarta) | 0.689085749 | 12 |  |
| hiv-1 nef: negative effector of fas and tnf(BioCarta) | 0.700507569 | 40 |  |
| er associated degradation (erad) pathway(BioCarta) | 0.720746193 | 12 |  |
| Circadian rhythm pathway(NCI/Nature) | 0.731979698 | 18 |  |
| Alpha-synuclein signaling(NCI/Nature) | 0.737291798 | 22 |  |
| Cell cycle(Kegg) | 0.789667306 | 32 |  |
| Canonical Wnt signaling pathway(NCI/Nature) | 0.819244193 | 27 |  |
| IL12-mediated signaling events(NCI/Nature) | 0.868288532 | 30 |  |
| Valine, leucine and isoleucine degradation(Kegg) | 0.887419876 | 15 |  |
| Toll-like receptor signaling pathway(Kegg) | 0.894024802 | 39 |  |
| sumoylation by ranbp2 regulates transcriptional repression(BioCarta) | 0.89816961 | 14 |  |
| EPO signaling pathway(NCI/Nature) | 0.92691644 | 36 |  |
| Signaling events mediated by HDAC Class I(NCI/Nature) | 0.938428024 | 53 |  |
| Sumoylation by RanBP2 regulates transcriptional repression(NCI/Nature) | 0.969477938 | 16 |  |
| wnt signaling pathway(BioCarta) | 0.970590778 | 34 |  |
| Presenilin action in Notch and Wnt signaling(NCI/Nature) | 0.975012369 | 32 |  |
| Apoptosis(Kegg) | 0.97779474 | 28 |  |
| inactivation of gsk3 by akt causes accumulation of b-catenin in alveolar macrophages(BioCarta) | 0.980455544 | 36 |  |
| IL27-mediated signaling events(NCI/Nature) | 0.989545942 | 20 |  |
| cell cycle: g1/s check point(BioCarta) | 0.996002582 | 23 |  |
| multi-step regulation of transcription by pitx2(BioCarta) | 0.996319181 | 28 |  |
| regulation of eif2(BioCarta) | 0.996893852 | 10 |  |
| mTOR signaling pathway(NCI/Nature) | 0.997296413 | 23 |  |
| Signaling events mediated by HDAC Class II(NCI/Nature) | 0.998326872 | 38 |  |
| segmentation clock(BioCarta) | 0.999486903 | 26 |  |
