# Supplementary material for: Detecting Cancer Gene Networks Characterized by Recurrent Genomic Alterations in a Population
Source: PLoS One. 2011 Jan 4;6(1):e14437. doi: 10.1371/journal.pone.0014437 (PMC3014942; doi:10.1371/journal.pone.0014437)
Supplement: Table S3 — , presented here, shows all pathways that found to be significant using Kaplan-Meier survival analysis. All of the pathways presented here were found to be significantly targeted through copy number alteration using the Fisher Omnibus test (after correction). All 29 pathways were tested in two more public datasets obtain from GEO (http://www.ncbi.nlm.nih.gov/geo). A - activity, C - consistency. (0.05 MB DOC) [file pone.0014437.s003.doc]

| Pathway Name | Fisher Omnibus P-Value | P-value for survival stratification using activity/ consistency  (Chin's Data) | P-value for survival stratification using activity/consistency  (GSE2990) | P-value for survival stratification using activity/consistency  (GSE3494) |
| --- | --- | --- | --- | --- |
| cdc25 and chk1 regulatory pathway in response to dna damage (BioCarta) | 2.22E-07 | 0.041(A) | 0.025(A) | 0.034(A)  0.005(C) |
| nfat and hypertrophy of the heart (BioCarta) | 1.72E-06 | 0.033(A) | 0.002(C) | 0.036(C) |
| Ras signaling in the CD4+ TCR pathway (NCI/Nature) | 1.62E-09 | 0.035(A) | 0.024(A)  0.03(C) | 0.01(A)  0.003(C) |
| Aurora A signaling (NCI/Nature) | 6.35E-07 | 0.03(A) | 0.013(A)  0.008(C) | 0.044(A)  0.025(C) |
| role of brca1 brca2 and atr in cancer susceptibility (BioCarta) | 1.28E-07 | 0.013(C) | 0.029(A) |  |
| a6b1 and a6b4 Integrin signaling  (NCI/Nature) | 1.19E-14 | 0.021(C) |  |  |
| agrin in postsynaptic differentiation  (BioCarta) | 1.88E-06 | 0.001(A) |  |  |
| alpha-synuclein and parkin-mediated proteolysis in parkinson`s disease (BioCarta) | 0 | 0.04(C) |  |  |
| angiotensin ii mediated activation of jnk pathway via pyk2 dependent signaling (BioCarta) | 5.07E-13 | 0.032(C) |  | 0.04(C) |
| Butanoate metabolism(Kegg) | 2.32E-08 | 0.02(A)  0.026(C) |  |  |
| ccr3 signaling in eosinophils (BioCarta) | 1.71E-11 | 0.02(A) |  |  |
| cyclin e destruction pathway (BioCarta) | 9.22E-09 | 0.005(A) |  |  |
| d4gdi signaling pathway (BioCarta) | 3.01E-11 | 0.029(C) |  |  |
| deregulation of cdk5 in alzheimers disease (BioCarta) | 0 | 0.027(A) |  | 0(C) |
| EGF signaling pathway (BioCarta) | 6.20E-07 | 0.032(A) | 0.029(C) |  |
| Endometrial cancer (Kegg) | 2.58E-09 | 0.029(C) |  |  |
| EphrinA-EPHA pathway (NCI/Nature) | 0 | 0.025(A) |  |  |
| Epithelial cell signaling in Helicobacter pylori infection (Kegg) | 2.83E-10 | 0.003(C) |  |  |
| Glycosaminoglycan degradation (Kegg) | 1.09E-08 | 0(A) |  |  |
| Glypican pathway (NCI/Nature) | 0 | 0.017(A) |  |  |
| Hypoxic and oxygen homeostasis regulation of HIF-1-alpha (NCI/Nature) | 1.48E-07 | 0(C) |  |  |
| IL4-mediated signaling events (NCI/Nature) | 7.94E-13 | 0.032(A) |  |  |
| induction of apoptosis through dr3 and dr4/5 death receptors (BioCarta) | 1.39E-09 | 0.017(C) |  |  |
| insulin signaling pathway (BioCarta) | 7.81E-06 | 0.012(A) | 0.01(A) |  |
| Novobiocin biosynthesis (Kegg) | 0 | 0.02(C) |  | 0.015(A)  0.004(C) |
| Phenylalanine, tyrosine and tryptophan biosynthesis (Kegg) | 0 | 0.04(C) |  | 0.04(A)  0.04(C) |
| pten dependent cell cycle arrest and apoptosis (BioCarta) | 0 | 0.016(C) | 0.023(A) |  |
| regulation of pgc-1a (BioCarta) | 3.41E-06 | 0.013(A) |  |  |
| Trk receptor signaling mediated by the MAPK pathway (NCI/Nature) | 7.65E-08 | 0.033(A) |  |  |
